# Utility of the ROX Index in Predicting Intubation for Patients With COVID-19–Related Hypoxemic Respiratory Failure Receiving High-Flow Nasal Therapy: Retrospective Cohort Study

**DOI:** 10.2196/29062

**Published:** 2021-08-27

**Authors:** Maulin Patel, Junad Chowdhury, Nicole Mills, Robert Marron, Andrew Gangemi, Zachariah Dorey-Stein, Ibraheem Yousef, Matthew Zheng, Lauren Tragesser, Julie Giurintano, Rohit Gupta, Parth Rali, Gilbert D'Alonzo, Huaqing Zhao, Nicole Patlakh, Nathaniel Marchetti, Gerard Criner, Matthew Gordon

**Affiliations:** 1 Department of Thoracic Medicine and Surgery Temple University Hospital Philadelphia, PA United States

**Keywords:** respiratory, medicine, nasal therapy, COVID-19, mechanical ventilation, ventilators, mortality, morbidity, intubation

## Abstract

**Background:**

The use of high-flow nasal therapy (HFNT) to treat COVID-19 pneumonia has been greatly debated around the world due to concerns about increased health care worker transmission and delays in invasive mechanical ventilation (IMV). Herein, we analyzed the utility of the noninvasive ROX (ratio of oxygen saturation) index to predict the need for and timing of IMV.

**Objective:**

This study aimed to assess whether the ROX index can be a useful score to predict intubation and IMV in patients receiving HFNT as treatment for COVID-19–related hypoxemic respiratory failure.

**Methods:**

This is a retrospective cohort analysis of 129 consecutive patients with COVID-19 admitted to Temple University Hospital in Philadelphia, PA, from March 10, 2020, to May 17, 2020. This is a single-center study conducted in designated COVID-19 units (intensive care unit and other wards) at Temple University Hospital. Patients with moderate and severe hypoxemic respiratory failure treated with HFNT were included in the study. HFNT patients were divided into two groups: HFNT only and intubation (ie, patients who progressed from HFNT to IMV). The primary outcome was the value of the ROX index in predicting the need for IMV. Secondary outcomes were mortality, rate of intubation, length of stay, and rate of nosocomial infections in a cohort treated initially with HFNT.

**Results:**

Of the 837 patients with COVID-19, 129 met the inclusion criteria. The mean age was 60.8 (SD 13.6) years, mean BMI was 32.6 (SD 8) kg/m², 58 (45%) were female, 72 (55.8%) were African American, 40 (31%) were Hispanic, and 48 (37.2%) were nonsmokers. The mean time to intubation was 2.5 (SD 3.3) days. An ROX index value of less than 5 at HFNT initiation was suggestive of progression to IMV (odds ratio [OR] 2.137, *P*=.052). Any further decrease in ROX index value after HFNT initiation was predictive of intubation (OR 14.67, *P*<.001). Mortality was 11.2% (n=10) in the HFNT-only group versus 47.5% (n=19) in the intubation group (*P*<.001). Mortality and need for pulmonary vasodilators were higher in the intubation group.

**Conclusions:**

The ROX index helps decide which patients need IMV and may limit eventual morbidity and mortality associated with the progression to IMV.

## Introduction

December 2019 was marked by a cluster of acute respiratory illnesses now known as COVID-19, caused by the novel coronavirus SARS-CoV-2. The virus has infected more than 8.7 million people worldwide with more than 460,000 reported deaths, resulting in a worldwide health care crisis [1,2]. The majority of morbidity from COVID-19 seems to arise from severe hypoxemic respiratory failure. As the pandemic spreads to the farthest reaches of the globe, health care centers have become overwhelmed, quickly exhausting their supply of ventilators and personnel who are trained to manage these critically ill patients. There is ongoing controversy concerning the optimal mode of respiratory support to treat COVID-19–associated hypoxemic respiratory failure.

The timing and adequacy of noninvasive forms of oxygen support (ie, high-flow nasal therapy [HFNT], simple face mask usage, etc) versus invasive mechanical ventilation (IMV) is not known. IMV has been associated with significant morbidity and mortality. In some case series, a mortality rate greater than 90% has been reported [3-6]. Case series from China, Italy, and New York, United States, have reported intubation rates ranging from 20.2% to 88% [4,6-9]. Early utilization of IMV has been greatly influenced by concerns for viral aerosolization and subsequently health care transmission through the use of noninvasive forms of oxygen support [10]. In addition, rapid progression of hypoxemic respiratory failure from mild dyspnea to acute respiratory distress syndrome (ARDS) within 48 to 72 hours has been noted in early studies [9,11]. Consequently, some centers decided to preemptively intubate patients with oxygen requirements as low as 6 L/min via nasal cannula for prolonged periods [3].

HFNT, in contrast to IMV, is a noninvasive oxygen system that delivers humidified air-oxygen blends and a titratable fraction of inspired oxygen (FiO_2_) as high as 60 L/min and 100% FiO_2_, respectively. Despite proven efficacy in other disease processes, the utilization of HFNT has been limited, and its use has not been widely recommended for patients with COVID-19–related pneumonia and hypoxemic respiratory failure. Limitations to the adoption of this mode of high-flow oxygenation include concerns about the rapid progression of the disease as well as fear of the aerosolization of SARS-CoV-2, resulting in increased transmission to health care providers [12-14].

However, HFNT has been successfully used in severe viral respiratory illnesses, including influenza A and H1N1 [15]. HFNT reduces the need for IMV rates compared to other modalities, with some studies also showing reduced 90-day mortality rates [16-19]. By decreasing the incidence of invasive ventilation, HFNT has the potential to decrease complications associated with IMV such as the incidence of ventilator-associated pneumonia.

Moreover, compared with noninvasive ventilation and conventional oxygen therapy, the use of HFNT has also been shown to reduce reintubation rates due to postextubation respiratory failure and has much better tolerability than noninvasive ventilation [20,21]. The Surviving Sepsis Guidelines for COVID-19 also recommends using HFNT in patients with acute hypoxemic respiratory failure due to COVID-19 [22].

The ROX index, defined as the ratio of oxygen saturation as measured by pulse oximetry (SpO_2_)/FiO_2_ to respiratory rate (RR) in breaths per minute, is a validated measurement that predicts outcomes when using HFNT to treat hypoxemic respiratory failure. An ROX index <4.88 after 12 hours predicts the need for IMV in patients with pneumonia [23].

Herein, we analyzed the utility of the ROX index to predict the need for and timing of IMV in a retrospective analysis of 129 patients with COVID-19–associated, moderate to severe hypoxemic respiratory failure treated with HFNT. In addition, mortality and rates of intubation, length of stay, and nosocomial infection in the cohort treated with HFNT are also reported.

## Methods

### Ethical Approval and Consent to Participate

The study was approved by the Temple University Institutional Review Board (TU-IRB protocol number: 27051). A waiver of consent was granted due to the acknowledged minimal risk to the patients.

### Patient and Public Involvement

Neither patients nor the public was involved in the design, conduct, reporting, or dissemination plans of our research.

### Design

A retrospective observation study of 1397 consecutive patients admitted to Temple University Hospital in Philadelphia, PA, from March 10, 2020, to May 17, 2020, was performed. Initial screening included patients who were either positive for COVID-19 using nasopharyngeal real-time reverse transcriptase–polymerase chain reaction (RT-PCR) or had high clinical suspicion based on high-resolution computerized tomography (HRCT) of the chest (typical peripheral nodular or ground-glass opacities without alternative cause) [24] with a typical inflammatory biomarker profile, but had a negative RT-PCR.

Thereafter, only patients with moderate and severe hypoxemic respiratory failure who were treated with HFNT at any point during their hospitalization were included in the study. Moderate and severe hypoxemic respiratory failure was defined as hypoxemia requiring more than 6 L/min of oxygen via nasal cannula. Absence of HFNT use during hospitalization was an exclusion criterion. Treatment protocols used at our hospital are described in Multimedia Appendix 1.

### Data Collection

Demographics, including age, sex, comorbidities, BMI, and smoking status (current smoker, nonsmoker), were collected. In addition, laboratory biomarkers on admission, including complete blood count with differential, ferritin, fibrinogen, lactate dehydrogenase (LDH), D-dimer, and C-reactive protein (CRP), were analyzed.

Respiratory metrics at the initiation of HFNT included RR, pulse oximetry, and FiO_2_. The same parameters were collected on days 1, 2, 3, and 5 after HFNT initiation. Parameters were recorded at the lowest FiO_2_ and highest pulse oximetry reported for the day. For patients who required IMV prior to the conclusion of data collection, respiratory parameters on the day of intubation were reported. Days on HFNT therapy, time to intubation (in days), average flow rate on HFNT, and the presence of hospital-acquired pneumonia or ventilator-associated pneumonia were also collected.

### Outcomes

The primary outcome was the ability of the ROX index to predict the need for IMV. Secondary outcomes included mortality, hospital length of stay, and hospital- or ventilator-acquired pneumonia. Hospital- and ventilator-acquired pneumonia was defined based on the presence of sputum positivity and treatment with antibiotics.

### Data Analysis

Our patients were divided into two groups for analysis: (1) HFNT support as a bridge to recovery (HFNT group) and (2) HFNT with progression to IMV (ie, intubation group). Comparisons were made between demographics, baseline laboratory values, and outcomes within the two groups. Changes in ROX index and concomitant changes in the clinical parameters of heart rate were also analyzed.

A multivariable prediction model for intubation for our cohort based on the above parameters was created. ROX index, comorbidities, and clinical and laboratory data were used to identify parameters that could predict the need for intubation. A receiver operating characteristic (ROC) curve was generated to determine the accuracy of the model.

### Statistical Methods

Continuous variables are presented as mean (SD) and categorical variables as counts and percentages. Continuous variables were compared with the use of the two-sample *t* test or the paired *t* test for categorical variables using the Pearson chi-square test. Laboratory data were nonparametric and were compared using the Wilcox rank-sum test. Kaplan-Meier analysis was estimated for survival and compared by the log-rank test.

To build a predictive model for intubation, multivariable logistic regression was performed to determine the adjusted associations of the variables with intubation. The initial model included all the variables associated with intubation in univariate analyses for *P*<.10. The final model optimized the balance of the fewest variables with good predictive performance. Assessment of model performance was based on discrimination and calibration. Discrimination was evaluated using the C-statistic, which represents the area under the ROC curve (AUC), where higher values represent better discrimination. Calibration was assessed by the Hosmer-Lemeshow test, where a *P* value greater than .05 indicates adequate calibration.

All statistical tests were two-tailed, and *P*<.05 was considered to indicate statistical significance. All statistical analyses were performed using Stata 14.0 (StataCorp).

### Availability of Supporting Data

The supporting data will be made available upon request.

## Results

### Demographics

**Figure 1:**
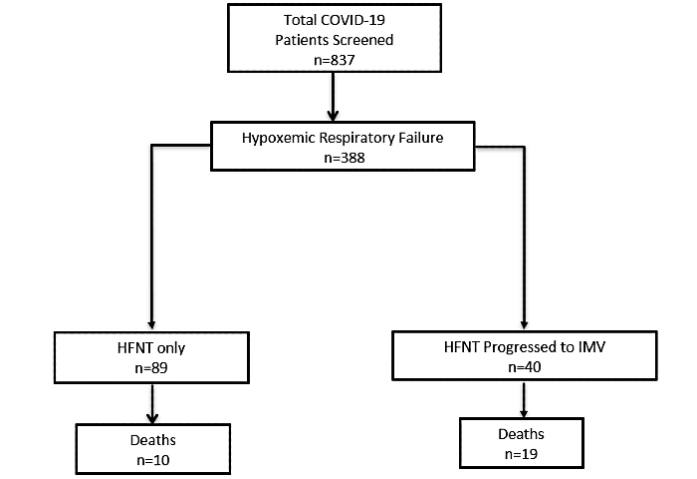
CONSORT (Consolidated Standards of Reporting Trials) diagram for screening. HFNT: high-flow nasal therapy, IMV: invasive mechanical ventilation.

A total of 1397 patients who were admitted to Temple University Hospital between March 10, 2020, and May 17, 2020, were screened. Of these, 837 patients had tested positive for COVID-19 by nasopharyngeal RT-PCR or were treated due to high clinical suspicion based on typical HRCT imaging and an inflammatory biomarker profile. Overall, 388 patients had hypoxemic respiratory failure, and 129 (15.4%) patients met our inclusion criteria of being on HFNT with moderate to severe hypoxemic respiratory failure (Figure 1). The mean age was 60.8 (SD 13.6) years, mean BMI was 32.6 (SD 8) kg/m², 58 (45 %) were female, 72 (55.8%) were African American, 40 (31%) were Hispanic, and 48 (37.2%) were nonsmokers. The major comorbidities reported (in descending incidence) were hypertension, diabetes, lung disease, heart disease, chronic kidney disease, malignancy, and psychiatric illness (Table 1). There were no differences in age, BMI, and gender between the groups. The proportion of nonsmokers was higher in the intubation group (22/40, 55% vs 26/89, 29.2%), as well as a trend toward a higher incidence of lung disease, chronic kidney disease, malignancy, and psychiatric disorders.

**Table 1:** Baseline demographics comparing the high-flow nasal therapy (HFNT) group and the intubation group (ie, HFNT with progression to invasive mechanical ventilation).

Characteristic			Total (N=129)	HFNT only (n=89)	Intubation (n=40)	*P* value
**Demographics**						
	Age (years), mean (SD)		60.8 (13.6)	60.7 (14.0)	61.2 (12.9)	.86
	BMI (kg/m^2^), mean (SD)		32.6 (8.0)	32.7 (8.0)	32.3 (8.0)	.80
	**Gender, n (%)**					.25
		Female	58 (45.0)	43 (48.3)	15 (37.5)	
		Male	71 (55.0)	46 (51.7)	25 (62.5)	
	**Race, n (%)**					.09
		African American	72 (55.8)	51 (57.3)	21 (52.5)	
		Caucasian	12 (9.3)	5 (5.6)	7 (17.5)	
		Hispanic	40 (31.0)	28 (31.5)	12 (30.0)	
		Other/unknown	5 (3.9)	5 (5.6)	0 (0)	
	**Smoking status, n (%)**					.006
		Smoking	72 (55.8)	58 (65.2)	14 (35.0)	
		Nonsmoker	48 (37.2)	26 (29.2)	22 (55.0)	
		Smoker	9 (7.0)	5 (5.6)	4 (10.0)	
		Unknown	—^a^	—	—	
	**Comorbidities, n (%)**					
		Lung disease	38 (29.7)	23 (26.1)	15 (37.5)	.19
		Hypertension	85 (65.9)	59 (66.3)	26 (65.0)	.89
		Heart disease	33 (25.6)	22 (24.7)	11 (27.5)	.74
		Diabetes mellitus	59 (45.7)	44 (49.4)	15 (37.5)	.21
		Chronic kidney disease	23 (17.8)	13 (14.6)	10 (25.0)	.15
		Psychiatric illness	10 (7.9)	4 (4.6)	6 (15.0)	.04
		Malignancy	15 (11.7)	4 (4.5)	11 (27.5)	<.001
	**Treatments, n (%)**					
		Remdesivir	11 (8.5)	7 (7.9)	4 (10.0)	.69
		Sarilumab	61 (47.3)	49 (55.1)	12 (30.0)	.008
		Anakinra	17 (13.2)	13 (14.6)	4 (10.0)	.47
		Tocilizumab	24 (18.6)	14 (15.7)	10 (25.0)	.21
		Etoposide	2 (1.6)	0 (0)	2 (5.0)	.03
		Intravenous immunoglobulin	38 (29.5)	21 (23.6)	17 (42.5)	.03
		Pulse steroids	111 (86.0)	75 (84.3)	36 (90.0)	.39
		Hydroxychloroquine	11 (8.5)	6 (6.7)	5 (12.5)	.28
		Gimsilumab	13 (10.1)	7 (7.9)	6 (15.0)	.21
		Plasma	15 (11.6)	9 (10.1)	6 (15.0)	.42
		Azithromycin	73 (70.2)	53 (73.6)	20 (62.5)	.25
	**Admission laboratory markers, mean (SD)**					
		Ferritin (ng/ml)	1193.5 (2490.9)	939.8 (1232.6)	1751.8 (4043.6)	.22
		C-reactive protein (mg/dl)	11.4 (8.0)	10.9 (7.4)	12.5 (9.0)	.30
		Lactate dehydrogenase (U/L)	425.3 (254.7)	401.4 (255.4)	478.5 (248.2)	.11
		D-dimer (ng/ml)	4719.6 (14,244.6)	3465.7 (10,618.9)	7509.5 (19,998.8)	.23
		Fibrinogen (mg/dl)	519.6 (185.0)	532.1 (158.1)	492.5 (233.4)	.34
		Absolute lymphocyte count (K/mm^3^)	1.4 (2.9)	1.1 (0.8)	2.2 (5.1)	.17
		Interleukin 6 (pg/ml)	743.1 (5026.8)	34.7 (45.9)	1634.2 (7526.2)	.25
		Interleukin 1 (pg/ml)	2.6 (3.9)	2.1 (0.5)	3.4 (6.0)	.30
		Aspartate aminotransferase (U/L)	58.5 (71.9)	49.5 (35.3)	79.0 (117.1)	.14
		Alanine aminotransferase (U/L)	41.8 (35.0)	40.0 (24.2)	45.9 (51.8)	.49
		Total bilirubin	0.9 (1.1)	0.8 (1.1)	1.2 (0.9)	.03
		Platelet (K/mm^3^)	215.4 (97.9)	219.5 (103.2)	206.1 (84.9)	.48
		Blood urea nitrogen (mg/dl)	29.1 (26.2)	26.3 (24.9)	35.3 (28.2)	.07
		Creatinine (mg/dl)	2.5 (3.9)	2.1 (3.9)	3.3 (3.8)	<.001
		Glomerular filtration rate (ml/min)	60.1 (32.7)	66.6 (31.6)	45.4 (30.5)	<.001
		Triglycerides (mg/dl)	166.7 (165.1)	143.2 (87.0)	213.8 (253.8)	.10
	**Respiratory parameters, mean (SD)**					
		HFNT use (days)	5.6 (5.1)	6.6 (5.5)	3.2 (3.1)	<.001
		HFNT flow rate	33.5 (11.7)	31.5 (9.7)	38.2 (14.6)	.012
		S/F^b^ ratio at admission	294.7 (131.6)	313.3 (125.6)	252.2 (136.8)	.015
		S/F at HFNT initiation	121.1 (38.4)	124.4 (38.8)	113.8 (37)	.15
		ROX^c^ at HFNT initiation	5.1 (2)	5.4 (2.1)	4.5 (1.6)	.02
		Pulmonary vasodilators	46 (35.7)	25 (28.1)	21 (52.5)	.007
		Ventilator use (days)	10.2 (7.6)	—	10.2 (7.6)	—
		Tracheostomy	11 (27.5)	—	11 (27.5)	—

^a^Not applicable.

^b^S/F: SpO_2_/FiO_2_ ratio.

^c^ROX: ratio of oxygen saturation.

### Treatments

Azithromycin (n=73, 70.2%) and steroids (n=111, 86%) were the most frequently utilized therapies. Immunomodulator therapy, including sarilumab, anakinra, intravenous immunoglobulin, and tocilizumab, was the next most commonly used therapies. There was a higher usage of gimsilumab, hydroxychloroquine, intravenous immunoglobulin, tocilizumab, and etoposide in the intubation group, while azithromycin was higher in the HFNT-only group. Steroid usage and other immunomodulators were similar across the groups.

### Laboratory Markers

Elevated inflammatory markers (ie, ferritin, CRP, D-dimer, fibrinogen, LDH, interleukin 6 [IL-6]), transaminitis, and lymphopenia were observed in all patients. There was a trend toward higher inflammatory markers (ie, ferritin, CRP, LDH, D-dimer, IL-6, interleukin 1), triglycerides, and transaminases in the intubation group. Significantly higher creatinine and lower glomerular filtration rate (GFR) were seen in the intubation group.

### Respiratory Parameters

The mean S/F (SpO_2_/FiO_2_) ratio at admission was 294.7 (SD 131.6) and was statistically different between the groups (mean 313.3, SD 125.6 vs mean 252.2, SD 136.8). The S/F ratio at high flow initiation was 121.1 (SD 38.4) overall, with no statistically significant differences between the groups (HFNT group: mean 124.4, SD 38.8 vs intubation group: mean 113.8, SD 37). The mean corresponding P/F (PaO_2_/FiO_2_) ratio at the start of HFNT was ~100.

Initial HFNT settings were 33.5 (SD 11.7) L/min of flow, while FiO_2_ was 84.1% (SD 20.3%). The intubation group had a statistically higher flow rate than the HFNT group. The average use of HFNT in our population was 5.6 (SD 5.1) days. The minimum settings on HFNT were 10-L flow and a FiO_2_ of 30%, while the maximum settings were 60-L flow and a FiO_2_ of 100%. The major complication with the use of HFNT was progression to IMV, which was seen in 40 (31.0%) patients. Average ventilator use in days was 10.2 (SD 7.6), and 10 (27.5%) patients received a tracheostomy. Overall, 46 (35.7%) patients required pulmonary vasodilators, with statistically higher usage in the intubation group.

### Outcomes

#### ROX Index Trends

**Figure 2:**
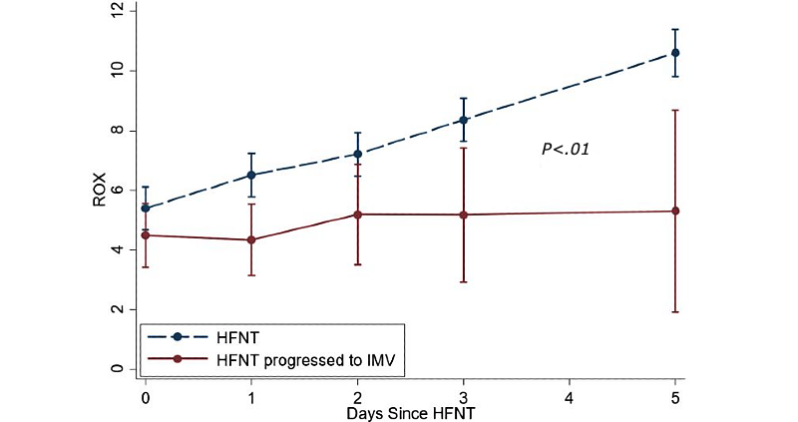
Average ROX (ratio of oxygen saturation) index progression of the high-flow nasal therapy (HFNT) group and the intubation group (ie, HFNT with progression to invasive mechanical ventilation [IMV]).

The mean ROX index value for the total cohort was 5.1 (SD 2.0) at HFNT initiation, and 5.9 (SD 2.5), 6.9 (SD 3.9), 8.1 (SD 4.1), and 10.3 (SD 5.9) on days 1, 2, 3, and 5, respectively. The mean ROX index consistently improved from initiation to day 5 in the HFNT group, while staying nearly constant in the intubation group (Figure 2). At each time interval, the ROX index was significantly higher in the HFNT group compared to the intubation group. The ROX change per day was also statistically different between the groups (HFNT group: mean 1.2, SD 1.3 vs intubation group: mean 0.3, SD 1.2). The ROX before intubation was lowest at 3.4 (SD 1.0) (Table 2).

**Table 2:** ROX (ratio of oxygen saturation) trends comparing the high-flow nasal therapy (HFNT) group and the intubation group (ie, HFNT with progression to invasive mechanical ventilation [IMV]).

Variable	Patients, N	Total ROX, mean (SD)	HFNT only, mean (SD)	Intubation, mean (SD)	*P* value
ROX at HFNT initiation	129	5.1 (2.0)	5.4 (2.1)	4.5 (1.6)	.02
ROX at day 1	119	5.9 (2.5)	6.5 (2.4)	4.3 (1.8)	<.001
ROX at day 2	101	6.9 (3.1)	7.2 (3.2)	5.2 (2.1)	.02
ROX at day 3	98	8.1 (4.1)	8.4 (4.2)	5.2 (1.9)	<.001
ROX at day 5	78	10.3 (5.9)	10.6 (5.9)	5.3 (2.0)	.08
ROX at IMV	40	3.4 (1.0)	—^a^	3.4 (1.0)	—
Mean ROX change per 24 hours	129	0.7 (1.5)	1.2 (1.3)	–0.3 (1.2)	<.001
ROX change per 24 hours	129	0.5 (0 to 1.5)^b^	1.2 (0.3 to 1.7) ^b^	0 (–0.5 to 0.1) ^b^	<.001

^a^Not applicable.

^b^Median (IQR).

#### Secondary Outcomes

**Figure 3:**
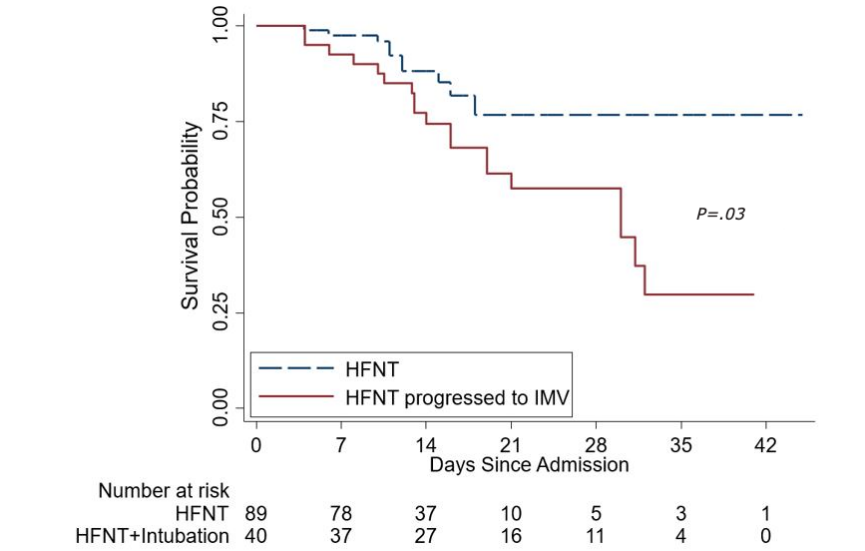
Kaplan-Meier comparing survival in the high-flow nasal therapy (HFNT) group and the intubation group (ie, HFNT with progression to invasive mechanical ventilation [IMV]).

Overall, mortality at our institution was 6.06% for patients positive for COVID-19 infection. However, in this cohort of severe hypoxemic respiratory failure, the mortality was 22.5% (n=29), with 11.2% (n=10) in the HFNT group and 47.5% (n=19) in the intubation group. Figure 3 shows the Kaplan-Meier curve between the two groups for survival. Of the 10 deaths in the HFNT group, 6 patients were in hospice care while the remaining were categorized as “do not resuscitate/intubate.” Average length of stay was statistically higher in the intubation group (HFNT group: 11.1 days vs intubation group: 19.5 days) (Table 3). The overall incidence of hospital-acquired pneumonia was significantly higher in the intubation group (25% [n=10] vs 1.1% [n=1], *P*<.001).

**Table 3:** Comparison of the high-flow nasal therapy (HFNT) group and the intubation group (ie, HFNT with progression to invasive mechanical ventilation [IMV]) for other outcomes.

Variable		Total (N=129)	HFNT only (n=89)	Intubation (n=40)	*P* value
**Days to IMV**					—^a^
	Mean (SD)	2.5 (3.3)	—	2.5 (3.3)	
	Median (IQR)	1 (1.0-3.0)	—	1 (1.0-3.0)	
Mortality, n (%)		29 (22.5)	10 (11.2)	19 (47.5)	<.001
Length of stay, mean (SD)		14.0 (8.0)	11.1 (4.7)	19.5 (9.9)	<.001
HAP^b^/VAP^c^, n (%)		11 (8.6)	1 (1.1)	10 (25.0)	<.001

^a^Not applicable.

^b^HAP: hospital-acquired pneumonia.

^c^VAP: ventilator-acquired pneumonia.

### Prediction Model

**Figure 4:**
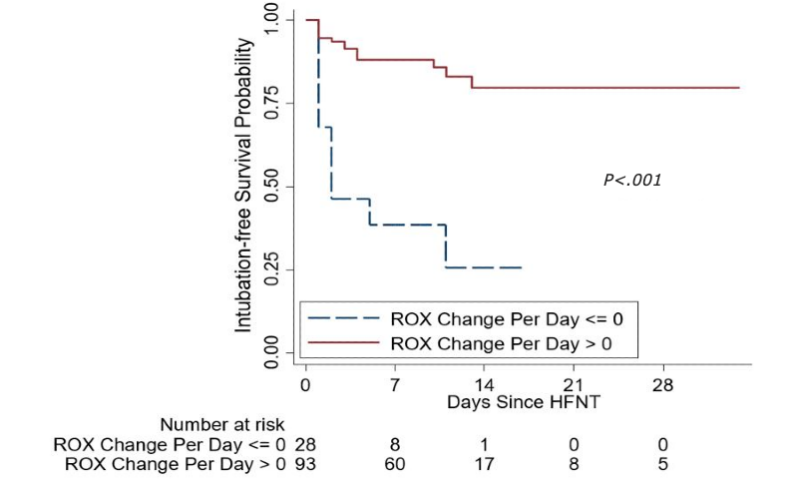
Kaplan-Meier estimator showing intubation-free survival probability by ROX (ratio of oxygen saturation) change per 24 hours. HFNT: high-flow nasal therapy.

**Figure 5:**
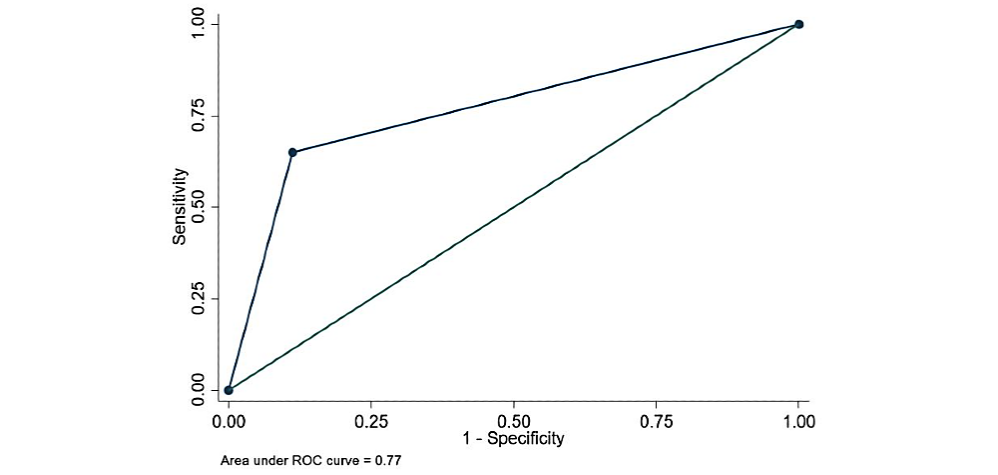
Receiver operating characteristic (ROC) curve predicting need for invasive mechanical ventilation using change in ROX (ratio of oxygen saturation) per 24 hours.

**Figure 6:**
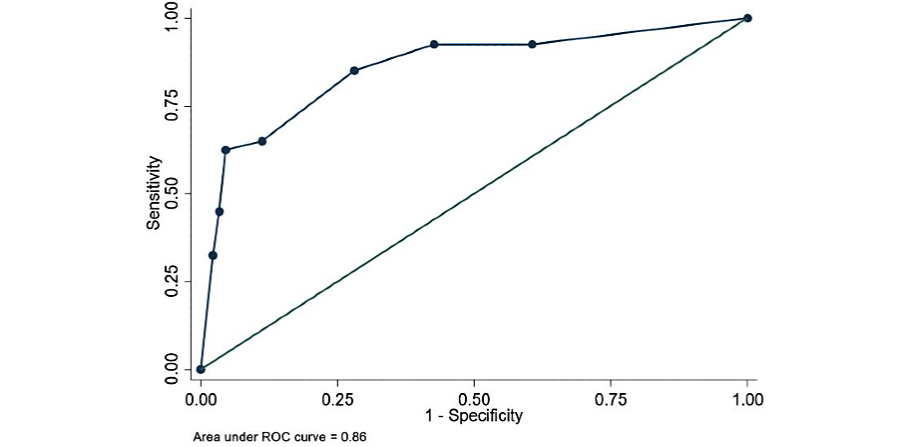
Receiver operating characteristic (ROC) curve of the multivariate model of change in ROX (ratio of oxygen saturation), D-dimer, and glomerular filtration rate to predict need for invasive mechanical ventilation.

At HFNC initiation, an ROX of <5 was nearly predictive of intubation (odds ratio [OR] 2.137, *P*<.06). Any change in ROX of less than or equal to zero (decrease or no change) after HFNT initiation over 24 hours was also predictive of intubation (OR 14.67, *P*<.001). A decrease in ROX by 1 over 24 hours regardless of the ROX index value was strongly predictive of intubation (OR 5, *P*<.001) (Table 4). Figure 4 shows intubation-free survival based on ROX change (≤0 versus >0) per 24 hours. In the univariate analysis, smoking, history of malignancy, admission LDH >500, peak D-dimer >4000, peak ferritin >1000, peak CRP ≥10, peak LDH >500, an ROX decrease as described above, admission triglycerides >200, and a GFR <60 were all predictive of intubation (Table S1, Multimedia Appendix 2). In the multivariate model, unchanged and/or decreased ROX over 24 hours, peak D-dimer >4000, and GFR <60 were predictive of intubation (Table 4). Figures 5 and 6 show the ROC curve for ROX change over 24 hours (AUC=0.77) and the multivariate model (AUC=0.86), respectively.

**Table 4:** A logistic regression model for predicting the need for invasive mechanical ventilation.^a^

Variable		Odds ratio		*P* value
**ROX^b^ at HFNT^c^ initiation**				.05
	≤5	2.137		
	>5	1		
**ΔROX from baseline (any 24-hour period)**				<.001
	Decreased by 1	5		
	Increased by 1	1		
**ΔROX change per day**				
	≤0	14.671	<.001	
	>0	1	<.001	
**Pulmonary vasodilators**				.008
	Yes	2.83		
	No	1		
**Final multivariate model**				
	ΔROX change per day (≤0 vs >0)	13.17	.001	
	Peak D-dimer (≥4000 vs <4000)	4.47	.003	
	GFR^d^ (≤60 vs >60)	3.29	.02	

^a^Univariate model in Multimedia Appendix 2.

^b^ROX: ratio of oxygen saturation.

^c^HFNT: high-flow nasal therapy.

^d^GFR: glomerular filtration rate.

## Discussion

### Principal Findings

In this retrospective review of patients with acute hypoxemic respiratory failure secondary to COVID-19 pneumonia, 129 patients were initially treated with HFNT. Out of this cohort, 89 patients remained on HFNT while 40 patients eventually required IMV. The 89 patients who were successfully treated with HFNT as a bridge to recovery experienced significant improvement in ROX from initiation of HFNT and at all recorded time points. In contrast, the ROX value for patients who ultimately required intubation remained steady or decreased over time. There were no associated deaths peri-intubation despite the presence of significant hypoxemia. There were no reported cases of failure to intubate resulting in an adverse outcome. Overall, the intubation group had a higher incidence of lung disease, chronic kidney disease, smoking, and malignancy.

HFNT is an important oxygen delivery modality that can help reduce intubation as seen by our overall institution intubation rate of 10%, which is significantly lower than what has been reported in the literature [4,6,7]. Moreover, there may be survival benefits to HFNT therapy among COVID-19 cases as seen in prior acute hypoxemic respiratory failure studies [13,25]. Despite our patient population having a higher incidence of lung disease and nicotine exposure than that reported in previous studies, the mortality rate was 22%, which is lower than prior reports [4,6,11].

Gattinoni and colleagues [26,27] proposed that patients with COVID-19 fall into two distinct groups or phenotypes. The “type L” or “non-ARDS type 1” phenotype has low elastance and high compliance. These patients often present with profound hypoxemia and low lung recruitability. The “type H” or “ARDS type 2” phenotype has high elastance and low compliance, requiring traditional management strategies of higher positive end-expiratory pressure (PEEP) and lower tidal volumes [26,27]. A significant number of patients with COVID-19 present with silent hypoxemia. As HFNT provides a modest PEEP effect (ie, 3-5 cm H_2_O at flow rates of 30-50 L/min with the mouth closed) [28], patients with predominant type L physiology may benefit from the oxygenation support that HFNC can provide noninvasively. HFNT also leads to a high oxygen reservoir by reducing anatomical dead space in the nasopharynx [29]. Often, higher tidal volumes are employed in the type L phenotype, which can lead to ventilator-associated lung injury (VILI). VILI can cause inflammatory cytokine release in patients with ARDS, including IL-6, both in critically ill people [30,31]. IL-6 in particular is one of the pathologic mechanisms for lung injury in COVID-19 [32,33]. Thus, the use of HFNT should not be overlooked in patients with severe COVID-19 respiratory failure.

Patient self-induced lung injury (P-SILI) has been cited as a theoretical contraindication to noninvasive methods of oxygenation. To date, however, P-SILI remains a conceptual model concept compared to VILI [34,35].

Optimal timing of IMV remains a point of debate, especially in patients previously supported with noninvasive forms of oxygen support, especially with regards to COVID-19. Based on our results, any decrease in the ROX index over a 24-hour period from baseline ROX at HFNT initiation is a strong predictor of intubation, irrespective of the total number of HFNT days. We chose to designate ROX change as ≤0 vs >0 for ease of use in the acute care setting.

Roca et al [23,36] previously used an ROX index of <4.8 at 12 hours to successfully identify patients at high risk for intubation among a cohort of 191 patients treated with HFNC for acute hypoxemic respiratory failure secondary to pneumonia [23,36]. Our analysis further supports their findings in the setting of viral pneumonia as opposed to predominantly bacterial pneumonia as was reported in their study. Our ROC analysis yielded similar results to initial studies. Thus, using serial measurements, we can identify patients on HFNT therapy in whom IMV should be considered based on changes in ROX [37].

Theoretically, the ROX can easily identify patients shifting from the type L to type H phenotype (lower S/F ratios and higher respiratory drive), thus minimizing subsequent risks of P-SILI. Another advantage of using the ROX index is its noninvasive nature based on readily available clinical parameters. The ROX index can be calculated remotely, thus preserving personal protective equipment and limiting health care exposure. When combined with a decreasing ROX index, a GFR <60 and D-dimer >4000 stratifies high-risk patients with increased accuracy. Kidney dysfunction makes patients susceptible to even small fluid shifts, thus worsening hypoxemia. D-dimer levels >4000 might possibly be a sign of microthrombi in pulmonary circulation, which has been described among COVID-19 cases [38].

Viral transmission through aerosolization by noninvasive forms of oxygenation such as HFNT remains controversial and is much debated. During the SARS (severe acute respiratory syndrome) outbreak in 2003, transmission to health care workers was reported in only 8% of HFNT patients [39]. This was demonstrated in further studies that proved that bacterial environmental contamination was not increased in the setting of HFNT use [40]. An in vitro study mimicking clinical scenarios including HFNT with mannequins only revealed proximal dispersion of secretions to the face and nasal cannula itself [41,42]. A recent study with healthy volunteers wearing high-flow nasal cannulas at both 30 L/min and 60 L/min of gas flow did not report variable aerosolization of particles between 10 to 10,000 nm, regardless of coughing, when compared with patients on room air or oxygen via regular nasal cannula [43]. At an institution with dedicated COVID-19 wards, only 1 of 80 staff members in our department had suspicion of health care transmission while directly caring for patients with COVID-19, thus re-emphasizing that HFNT did not present an increased risk of transmission to health care personnel.

### Strengths and Limitations

Our study has several strengths. It is the largest reported cohort utilizing HFNT in patients with COVID-19 thus far. The ROX index was able to successfully predict bridge to recovery or progression to IMV without demonstrable adverse effects from delaying the implementation of mechanical ventilation. In a high-risk, urban population with multiple comorbidities, the use of HFNT resulted in a lower rate of intubation, and suggests a possible mortality benefit while maintaining a low risk of health care transmission.

Our study has several limitations as well. First, it is a retrospective review, thus making it susceptible to unintended biases. However, developing a prospective study during a pandemic situation was impractical. Second, although this is the largest HFNT study, the total sample size is limited and representative of a single center’s experience. Lastly, we were unable to provide consistent details on the presence and degree of hypercapnia for our cohort due to our institutional policy to minimize staff exposure to COVID-19 infection.

### Conclusion

In conclusion, the ROX index serves as an accurate risk stratification tool in patients with moderate and severe hypoxemic respiratory failure secondary to COVID-19 pneumonia. HFNT can be safely and successfully implemented while utilizing the ROX index to predict the need for IMV. Monitoring ROX trends may allow clinicians to avoid any significant delays in escalating the level of care or implementing IMV. The use of HFNT not only reduces intubation rates but also has the potential to reduce mortality and morbidity associated with IMV.

## Appendix

Multimedia Appendix 1 Treatment protocols.

Multimedia Appendix 2 Univariate analysis predicting the need for invasive mechanical ventilation.

Multimedia Appendix 3 List of Temple University Research Group collaborators.

## Abbreviations

ARDS: acute respiratory distress syndrome
AUC: area under the receiver operating characteristic curve
CRP: C-reactive protein
GFR: glomerular filtration rate
HFNT: high-flow nasal therapy
HRCT: high-resolution computerized tomography
IL-6: interleukin 6
IMV: invasive mechanical ventilation
LDH: lactate dehydrogenase
OR: odds ratio
P-SILI: patient self-induced lung injury
P/F: PaO_2_/FiO_2_ ratio
PEEP: positive end-expiratory pressure
ROC: receiver operating characteristic
ROX: ratio of oxygen saturation
RT-PCR: reverse transcriptase–polymerase chain reaction
S/F: SpO_2_/FiO_2_ ratio
SARS: severe acute respiratory syndrome
VILI: ventilator-associated lung injury
