# Supplementary material for: Utility of the ROX Index in Predicting Intubation for Patients With COVID-19–Related Hypoxemic Respiratory Failure Receiving High-Flow Nasal Therapy: Retrospective Cohort Study
Source: JMIRx Med. 2021 Aug 27;2(3):e29062. doi: 10.2196/29062 (PMC8404242; doi:10.2196/29062)
Supplement: Multimedia Appendix 1 [file xmed_v2i3e29062_app1.docx]

**Multimedia Appendix 1.** Treatment protocols.

HFNT was provided with a humidified air-oxygen blender starting at 35 LPM with the fraction of inspired oxygen (F_I_O_2_) adjusted to maintain oxygen saturations > 92%; further adjustments were made based on patients’ tolerance and goals of oxygenation. The initial temperature for the high flow setup was 37^0^ C and was titrated between 34-37^0^ C for patient comfort. As an institutional policy, HFNT was preferred over IMV and was maintained indefinitely as long as oxygenation, ventilation, and work of breathing parameters were acceptable. Data on initial oxygenation support included flow of air-oxygen blend in LPM and F_I_O_2_. The decision to switch to NIV or IMV was at the discretion of the clinical care team. Once on IMV, patients were assessed daily for appropriateness of spontaneous breathing trials (SBT) and spontaneous awakening trials (SAT) for extubation per standard guidelines. Patients were extubated following a SBT of 15 - 30 minutes on either pressure support ventilation (PSV) of 5cmH_2_O or T-piece with a viral filter based on clinical judgement and rapid shallow breathing index.

All patients admitted due to respiratory symptoms and classic radiographic evidence of COVID-19 pneumonia were admitted to a specialized hospital unit and given antibiotic therapy for community acquired pneumonia and systemic steroids with methylprednisolone at 0.5 to 1.0 mg/kg for at least 5-10 days. Patients with RT-PCR positive swabs were also screened for eligibility for randomized controlled trials at our institution which included sarilumab (Regeneron Pharmaceuticals; NCT04315298), remdesivir (Gilead Sciences; NCT04292730 and NCT04292899), gimsilumab (Kinevant Sciences: NCT04351243), and convalescent plasma (Mayo Clinic; NCT04338360). Those with significant disease, but ineligible for clinical trials were treated with compassionate use of anakinra, tocilizumab or etoposide based on institutional care pathways. Other therapies included high-dose corticosteroids (defined as a minimum daily 125 mg of methylprednisolone and above, regardless of bolus frequency), intravenous immunoglobulin (IVIG), and hydroxychloroquine (HCQ). Therapies were offered based on clinical severity, radiographic burden, and/or presence of cytokine storm as evidenced by inflammatory markers. The decision to select from these choices was made by the same institutional multidisciplinary team including pulmonologists and rheumatologists.
