# Supplementary material for: Utility of the ROX Index in Predicting Intubation for Patients With COVID-19–Related Hypoxemic Respiratory Failure Receiving High-Flow Nasal Therapy: Retrospective Cohort Study
Source: JMIRx Med. 2021 Aug 27;2(3):e29062. doi: 10.2196/29062 (PMC8404242; doi:10.2196/29062)
Supplement: Multimedia Appendix 2 [file xmed_v2i3e29062_app2.docx]

**Multimedia Appendix 2**. Univariate analysis predicting the need for invasive mechanical ventilation.

Table 1: Univariate Analysis Predicting Need for IMV

| **Variables** | **Odds Ratio** | **P-value** |
| --- | --- | --- |
| *Age*  <65  >=65 | 1  1.19 | 0.644 |
| *BMI*  <30  >=30 | 1  0.84 | 0.725 |
| *Smoker*  No  Yes | 1  3.5 | 0.006 |
| Heart Disease  Lung Disease  Diabetes  HTN  CKD  Malignancy | 1.15  1.69  0.61  0.94  1.94  8.06 | 0.738  0.192  0.208  0.886  0.154  0.0002 |
| *Admission Laboratory markers*  Ferritin (ng/ml) > 1000  CRP (mg/dl) >= 10  LDH (U/L) >= 500  D-dimer (ng/ml) >= 4000  Triglycerides (mg/dl) >=200  GFR (ml/min) < 60 | 1.44  0.95  2.74  1.39  3.03  3.77 | 0.378  0.905  0.016  0.549  0.015  0.0008 |
| *Peak Laboratory markers*  Ferritin (ng/ml) > 1000  CRP (mg/dl) >= 10  LDH (U/L) >= 500  D-dimer (ng/ml) >= 4000 | 3.0  4.13  5.8  4.93 | 0.005  0.009  <0.0001  <0.0001 |
| ROX at HFNT initiation  =<5  >5 | 2.137  1 | 0.0517 |
| ΔROX from baseline (any 24-hr period)  Decreased by 1  Increased by 1 | 5  1 | 0.0001 |
| ΔROX change per day  =< 0  > 0 | 14.671  1 | 0.0001 |
| Pulmonary Vasodilators  Yes  No | 2.83  1 | 0.0084 |
