# Supplementary material for: Utility of the ROX Index in Predicting Intubation for Patients With COVID-19–Related Hypoxemic Respiratory Failure Receiving High-Flow Nasal Therapy: Retrospective Cohort Study
Source: JMIRx Med. 2021 Aug 27;2(3):e29062. doi: 10.2196/29062 (PMC8404242; doi:10.2196/29062)
Supplement: Multimedia Appendix 3 [file xmed_v2i3e29062_app3.docx]

**Multimedia Appendix 3.** List of Temple University Research Group collaborators.

List of personnel for Temple University COVID-19 Research Group

**Name** **Department**

Aaron Mishkin Infectious Disease

Abbas Abbas TMS

Abhijit S Pathak Surgery

Abhinav Rastogi Admin

Adam Diamond Pharmacy

Aditi Satti TMS

Adria Simon Emergency Medicine

Ahmed Soliman TMS

Alan Braveman TMS

Albert J Mamary TMS

Aloknath Pandya TMS

Amy Goldberg Surgery

Amy Kambo TMS

Andrew Gangemi TMS

Anjali Vaidya Cardiology

Ann Davison TMS

Anuj Basil Cardiology

Bakhos, Charles T TMS

Bill Cornwell TMS

Brianna Sanguily TMS

Brittany Corso Internal Medicine

Carla Grabianowski TMS

Carly Sedlock Infectious Disease

Catherine Myers TMS

Catherine Myers TMS

Charles Bakhos TMS

Chenna Kesava Reddy Mandapati, TMS

Cherie Erkmen TMS

Chethan Gangireddy Cardiology

Chih-ru Lin TMS

Christopher T Burks Lab Administration

Claire Raab Internal Medicine

Crabbe, Deborah Cardiology

Crystal Chen Internal Medicine

Daniel Edmundowicz Cardiology

Daniel Sacher TMS

Daniel Salerno TMS

Daniele Simon Emergency Medicine

David Ambrose TMS

David Ciccolella TMS

Debra Gillman TMS

Dolores Fehrle TMS

Dominic Morano TMS

Donnalynn Bassler TMS

Edmund Cronin Cardiology

Eduardo Dominguez TMS

Ekam Randhawa TMS

Ekamjeet Randhawa TMS

Eman Hamad Cardiology

Eneida Male TMS

Erin Narewski TMS

Francis Cordova TMS

Frederic Jaffe TMS

Frederich Kueppers TMS

Fusun Dikengil TMS

Galli, Jonathan TMS

Gangemi, Andrew TMS

Garfield, Jamie TMS

Gayle Jones TMS

Gennaro Calendo TMS

Gerard Criner TMS

Gilbert D'Alonzo TMS

Ginny Marmolejos TMS

Gordon, Matthew TMS

Gregory Millio Internal Medicine

Gupta, Rohit TMS

Gustavo Fernandez TMS

Hannah Simborio TMS

Harwood Scott TMS

Heidi Shore-Brown TMS

Hernan Alvarado Respiratory Care

Ho-Man Yeung Internal Medicine

Ibraheem Yousef TMS

Ifeoma Oriaku TMS

Iris Jung-won Lee Nephrology

Isaac Whitman Cardiology

James Brown TMS

Jamie L. Garfield TMS

Janpreet Mokha TMS

Jason Gallagher School of Pharmacy

Jeffrey Stewart TMS

Jenna Murray TMS

Jessica Tang TMS

Jeyssa Gonzalez TMS

Jichuan Wu TMS

Jiji Thomas TMS

Jim Murrett Ultrasound Fellow

Joanna Beros TMS

John M. Travaline TMS

Jolly Varghese TMS

Jordan Senchak Internal Medicine

Joseph Lambert TMS

Joseph Ramzy TMS

Joshua Cooper Cardiology

Jun Song Medical Student

Junad Chowdhury TMS

Kaitlin Kennedy TMS

Karim B Ahmed TMS

Karim Loukmane TMS

Karthik Shenoy TMS

Kathleen Brennan TMS

Keith Johnson TMS

Kevin Carney TMS

Kraftin Schreyer Emergency Medicine

Kristin Criner Endo

Kumaran, Maruti Radiology

Lauren Miller TMS

Laurie Jameson TMS

Laurie Johnson TMS

Laurie Kilpatrick TMS

Lii-Yoong Criner TMS

Lily Zhang TMS

Lindsay K Mcgann Hospitalist

Llera A Samuels TMS

Marc Diamond TMS

Margaret Kerper TMS

Maria Vega Sanchez TMS

Mariola Marcinkienwicz TMS

Maritza Pedlar TMS

Mark Aksoy TMS

Mark Weir TMS

Marla R. Wolfson TMS

Marla Wolfson TMS

Marron, Robert TMS

Martin Keane Cardiology

Massa Zantah TMS

Mathew Zheng TMS

Matthew Delfiner Internal Medicine

Matthew Gordon TMS

Maulin Patel TMS

Megan Healy Emergency Medicine

Melinda Darnell TMS

Melinda Darnell TMS

Melissa Navaro TMS

Meredith A. Brisco-Bacik Cardiology

Michael Bromberg Hematology

Michael Gannon Cardiology

Michael Jacobs TMS

Mira Mandal TMS

Nanzhou Gou TMS

Narewski, Erin TMS

Nathaniel Marchetti TMS

Nathaniel Xander

TMS

Navjot Kaur TMS

Neil Nadpara Internal Medicine

Nicole Desai Internal Medicine

Nicole Mills TMS

Norihisa Shigemura Surgery

Ohoud Rehbini TMS

Oisin O'Corragain TMS

Oisin O'Corragain TMS

Omar Sheriff TMS

Oneida Arosarena Otolaryngology

Osheen Abramian TMS

Paige Stanley TMS

Parag Desai TMS

Parth Rali TMS

Patrick Mulhall Pulm

Pravin Patil Cardiology

Priju Varghese Internal Medicine

Puja Dubal TMS

Puja Patel TMS

Rachael Blair TMS

Rajagopalan Rengan TMS

Rami Alashram TMS

Randol Hooper TMS

Rebecca A Armbruster Chief Medical Officer

Regina Sheriden TMS

Robert Marron TMS

Roberto Caricchio Rheumatology

Rogers Thomas TMS

Rohit Gupta TMS

Rohit Soans Surgery

Roman Petrov TMS

Roman Prosniak TMS

Romulo Fajardo Surgery

Ruchi Bhutani TMS

Ryan Townsend TMS

Sabrina Islam Cardiology

Samantha Pettigrew Internal Medicine

Samantha Wallace TMS

Sameep Sehgal TMS

Samuel Krachman TMS

Santosh Dhungana TMS

Sarah Hoang TMS

Sean Duffy TMS

Seema Rani TMS

Shapiro William TMS

Sheila Weaver TMS

Shelu Benny TMS

Sheril George TMS

Shuang Sun TMS

Shubhra Srivastava-Malhotra TMS

Stephanie Brictson TMS

Stephanie Spivack Infectious Disease

Stephanie Tittaferrante Internal Medicine

Stephanie Yerkes TMS

Stephen Priest Internal Medicine

Steve Codella TMS

Steven G Kelsen TMS

Steven Houser Research

Steven Verga TMS

Sudhir Bolla TMS

Sudhir Kotnala TMS

Sunil Karhadkar Surgery

Sylvia Johnson TMS

Tahseen Shariff TMS

Tammy Jacobs TMS

Thomas Hooper TMS

Tom Rogers TMS

Tony S. Reed Chief Medical Officer

Tse-Shuen Ku TMS

Uma Sajjan TMS

Victor Kim TMS

Whitney Cabey Emergency Medicine

Wissam Chatila TMS

Wuyan Li TMS

Zach Dorey-Stein TMS

Zachariah Dorey-Stein TMS

Zachary D Repanshek Emergency Medicine

*TMS – Thoracic Medicine and Surgery
